# T-Cells Null for the MED23 Subunit of Mediator Express Decreased Levels of KLF2 and Inefficiently Populate the Peripheral Lymphoid Organs

**DOI:** 10.1371/journal.pone.0102076

**Published:** 2014-07-23

**Authors:** Lawryn H. Kasper, Tomofusa Fukuyama, Paul K. Brindle

**Affiliations:** Department of Biochemistry, St. Jude Children's Research Hospital, Memphis, Tennessee, United States of America; Institute of Genetics and Molecular and Cellular Biology, France

## Abstract

MED23, a subunit of the Mediator coactivator complex, is important for the expression of a subset of MAPK/ERK pathway-responsive genes, the constituents of which vary between cell types for reasons that are not completely clear. MAPK/ERK pathway-dependent processes are essential for T-cell development and function, but whether MED23 has a role in this context is unknown. We generated *Med23* conditional knockout mice and induced *Med23* deletion in early T-cell development using the lineage specific *Lck-Cre* transgene. While the total cell number and distribution of cell populations in the thymuses of *Med23^flox/flox^;Lck-Cre* mice were essentially normal, MED23 null T-cells failed to efficiently populate the peripheral lymphoid organs. MED23 null thymocytes displayed decreased expression of the MAPK/ERK-responsive genes *Egr1*, *Egr2*, as well as of the membrane glycoprotein *Cd52* (CAMPATH-1). MED23 null CD4 single-positive thymocytes also showed decreased expression of KLF2 (LKLF), a T-cell master regulatory transcription factor. Indeed, similarities between the phenotypes of mice lacking MED23 or KLF2 in T-cells suggest that KLF2 deficiency in MED23 null T-cells is one of their key defects. Mechanistic experiments using MED23 null MEFs further suggest that MED23 is required for full activity of the MAPK-responsive transcription factor MEF2, which has previously been shown to mediate *Klf2* expression. In summary, our data indicate that MED23 has critical roles in enabling T-cells to populate the peripheral lymphoid organs, possibly by potentiating MEF2-dependent expression of the T-cell transcription factor KLF2.

## Introduction

While certain histone N-terminal tail modifications and coactivator recruitment events correlate well with gene expression on a genome-wide scale, mutagenesis studies to test their roles directly have often produced unexpectedly modest or specific effects (reviewed in [1], [2]). This apparent paradox indicates that target gene context is a critical, but still poorly understood aspect of transcriptional regulation. Coactivator context specificity has been evident, for example, since early descriptions of yeast mutants that affect amino acid biosynthesis, mating type switching, and sucrose fermentation; phenotypes that were later ascribed to mutations in “global” coactivators (e.g. GCN5, SWI/SNF). In mammals, the problem of coactivator functional specificity has been illuminated by the use of mice and cells with domain-specific or tissue-specific conditional-null mutations in coactivator genes [3]–[14].

Multi-subunit coactivator complexes, such as Mediator, represent another dimension of the “context paradox.” The large Mediator complex, and its variants, interacts with RNA polymerase II and forms part of the general transcriptional machinery [15], yet mutation or knock-down of individual subunits in mice and cells have revealed curiously distinct phenotypes [16]. Indeed, it has previously been shown that expression of the same target gene can have a different requirement for the MED23 subunit in different cell types, even in response to the same signal [17]. These kinds of context-dependent functionalities of Mediator and its subunits are perplexing, and understanding how and why they occur remains a challenge [18].

MED23 (SUR2, DRIP130, CRSP3) is a ∼130 kDa subunit of Mediator that was initially identified in a screen for suppressors of an activated RAS induced phenotype in *C. elegans* where it was determined to act downstream in the RAF/MAPK pathway [19]. Studies of *Med23* knockout ES cells and MEFs confirm that MED23 loss affects the RTK-RAS-RAF-MEK-ERK axis, resulting in decreased serum-responsive gene expression and defective MAPK-dependent transactivation by ETS transcription factor family members, ELK1 and to a lesser degree, ETS1 and ELK4 (SAP1) [17],[20]. In line with these results, MED23 has been shown to be important in ELK1-dependent adipogenesis [21],[22], and the proliferation of non-small cell lung cancers with activating mutations in RAS [23].

The RTK-RAS-RAF-MEK-ERK axis of the MAPK pathway plays important roles in normal T-cell development and function that include signaling through the T-cell receptor (TCR), regulating thymocyte positive selection, and T-cell homeostasis [24]–[30]. While MAPK-dependent ETS family transcription factors that require MED23 for full transcriptional activity in MEFs or ES cells are necessary for normal T-cell development [31], cell type context clearly influences which target genes display MED23 dependence [17]. This made it unclear how MED23 deletion would impact T-cells. Using a T lineage specific knock out of *Med23*, we found that while MED23 null thymocyte numbers and proportions are essentially normal, the number of MED23 null T-cells in the periphery is reduced. We discovered a novel requirement for MED23 in the expression of KLF2, a T-cell master regulatory transcription factor [32]. Mechanistic experiments in MEFs suggest that MED23 is required by the transcription factor MEF2 to directly regulate *Klf2* expression.

## Materials and Methods

### Mice

*Med23* conditional knock out mice were generated by inserting LoxP sites into the introns flanking the region containing exons 5, 6 and 7 of *Med23* (encoding amino acids 96 to 199) using a transposon based system reported previously [11]. The *Lck-Cre* transgenic mice were originally reported in Hennet *et al.* [33]. For some experiments, mice also contained an eYFP reporter transgene that was expressed after Cre-mediated recombination [34]. All mouse experiments followed protocols approved by the St Jude Animal Care and Use Committee.

### Mouse embryonic fibroblasts (MEFs)

*Med23^flox/flox^* embryos homozygous for LoxP conditional alleles of *Med23* and wild type littermate control embryos were harvested at e14.5 to produce mouse embryonic fibroblasts (MEFs) that were maintained in 3% oxygen to delay the onset of senescence [35]. Primary MEFs were transduced with adenovirus expressing cre recombinase (MOI 100) for 16 h and experiments were performed four days after transduction. Recombination of the flox sites in *Med23* was confirmed by semi-quantitative genomic PCR and western blot.

### Genotyping

PCR to check genotype and allele recombination was carried out with a three primer reaction (Primer 1: ATTCATGGCCAACACAGCCC, Primer 2: GCCCAAAGCTGTGTTCTTTCCC and Primer 3: CACTGAGTGTGGCAGCTCATG) using Qiagen reagents including Q solution (Cat. # 201207) at 94°C 5 min followed by 30 cycles of (94°C 10 sec; 60°C 1 min; 68°C 2 min) with a final extension of 72°C 10 min and bands representing the wild type allele (∼1.1 kb), the LoxP flanked unrecombined allele (∼1.35 kb) and the recombined LoxP allele (∼1.8 kb) were resolved on a 1.2% agarose gel.

### Flow cytometry, FACS and MACS cell sorting

Flow cytometry was performed on BD Biosciences FACS Calibur and FACS LSR instruments. FACS of CD4^+^ or CD8^+^ single positive thymocytes was performed on a BD Biosciences Aria. All antibodies were from Becton Dickenson. For some experiments CD4^+^ single positive thymocytes were enriched using MACS biotin beads (Miltenyi) and anti-CD8 antibody (Miltenyi) to remove the CD8 expressing single positive and CD4^+^/CD8^+^ double positive thymocytes.

### Proliferation assay

Single cell suspensions of total thymocytes were seeded at 0.25×10^6^ cells per well of a 96 well plate. Anti-CD3 (10 µg/ml) and anti-CD28 (10 µg/ml) antibodies were immobilized in wells as noted by incubating for 90 min at 37°C, then the wells were washed three times with PBS prior to use. Cells were allowed to proliferate for 44 hrs at 37°C, then 1 µCi ^3^H thymidine was added to each well and cells were allowed to proliferate a further 18 hrs before the cells were harvested onto a filter, washed and counted.

### TUNEL assay

Frozen sections of thymus and spleen were fixed for 20 min at room temperature with 4% paraformaldehyde in PBS, then washed and incubated in PBS for an additional 30 min. Sections were permeabilized (0.1% Triton X-100/0.1% sodium citrate) for 2 min at 4°C, washed twice with PBS and incubated for 1 hr at 37°C with the TUNEL labeling mixture from the Roche In Situ Cell Death Detection Kit, TMR red (Cat. No. 12156792910). Slides were washed with PBS, stained with DAPI and imaged. As a positive control, one frozen section was incubated with 1 µg/ml DNaseI for 10 min at room temperature prior to TUNEL labeling.

### Gene expression

RNA was isolated using Trizol (Life Technologies). Microarray platforms used were Affymetrix Murine Genome U74A Version 2 Array and were analyzed using Spotfire software (TIBCO). RNA from thymocytes was isolated immediately following FACS or thymocytes were rested for 4 hrs after harvest then plated for 3 hrs with plate bound αCD3 antibody as indicated in figure legends. Array data were deposited with GEO (GSE 57061). Reverse transcriptase reactions were performed using Superscript II (Life Technologies). qPCR was performed on an MJ Research Opticon real time machines using Quantitect SYBR Master Mix (Qiagen).

### Antibodies

The KLF2 (LKLF) rabbit polyclonal antibody was provided by Jeffrey Leiden and Laurie Glimcher and was originally published in [36]. The MED23 rabbit polyclonal antibody was generated against a synthetic peptide corresponding to mouse MED23 residues 897–916 (based on human MED23 residue numbering) crosslinked to KLH. It is available from Rockland (DRIP130 antibody, Cat. # 100-401-239).

### Plasmids and transient transfection assays

The mouse −215 *Klf2* promoter construct (pGL3-*Klf2*-pro) and the GAL4-KLF2 construct containing amino acids 1–88 of mouse KLF2 fused to the GAL4 DNA binding domain were provided by Jerry Lingrel [37],[38]. The MEF2 site in the *Klf2* promoter reported in Kumar *et al.* [39] was mutated in the mouse pGL3-*Klf2*-pro (ctaAATTtag to ctaTCGGtag) to yield pGL3-*Klf2*-pro-mut. The GAL4-MEF2C construct was made using the pM1 GAL4 DBD containing vector backbone [40] (Clontech). The full length MEF2C cDNA was PCR amplified from a cDNA library made from Universal Mouse Reference RNA (Stratagene). Transient transfection assays using MEFs were performed as previously described using the Promega Dual Luciferase Reporter Assay Kit where reporter gene luciferase activity is normalized to cotransfected *Renilla* luciferase reporter activity [8].

## Results

### MED23 null T-cells develop normally, but are defective in populating the periphery

We generated mice bearing a conditional knockout allele of *Med23* by inserting LoxP sites in the introns flanking the region containing exons 5, 6 and 7 of *Med23* encoding amino acids 96 to 199 (Figure S1A). Cre-mediated recombination of the LoxP sites in the *Med23^flox^* allele would result in a frameshift if exons 4 and 8 spliced fortuitously. Using an *Lck-Cre* transgene that deletes during the DN4 stage of T-cell development [33],[41], we produced *Med23^flox/flox^;Lck-Cre* mice to assess the role of MED23 in T-cells. Efficient recombination of the *Med23^flox^* alleles in the thymuses of *Med23^flox/flox^;Lck-Cre* mice (Figure S1B) resulted in a near total loss of MED23 protein (Figure S1C).

**Figure 1:**
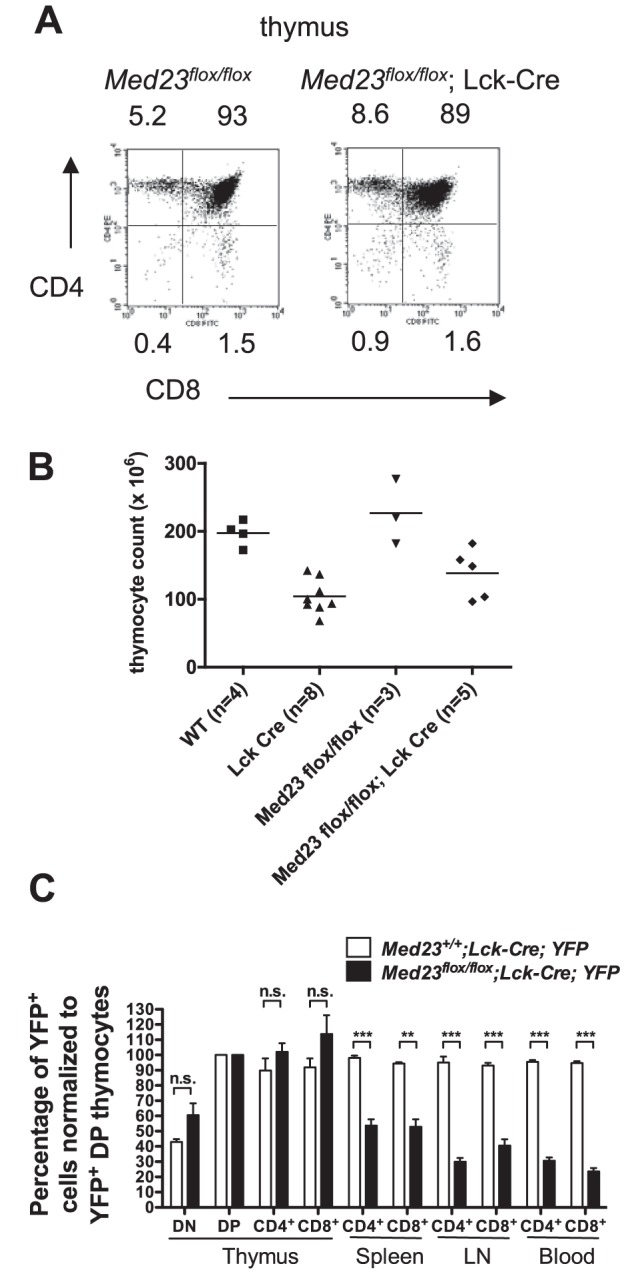
*Med23^flox/flox^*;*Lck-Cre* mice have normal thymic populations, but MED23 null T-cells poorly populate the peripheral lymphoid tissues. **A**, FACS analysis of thymus from *Med23^flox/flox^* (control) and *Med23^flox/flox^;Lck-Cre* mice showing CD4 and CD8 subpopulations. **B**, Total thymocyte counts from 5–8 week old mice. Note that the *Lck-Cre* transgene alone decreases the total number of thymocytes. **C**, Flow cytometry for deleted (YFP^+^) T-cells. Since mice have differing overall deletion efficiencies, deletion percentage for each T-cell type and compartment was normalized to the deletion in double positive (DP) thymocytes for each animal. *Med23^+/+^;Lck-Cre;YFP*, N = 2. *Med23^flox/flox^;Lck-Cre;YFP*, N = 7. Mean +/−SEM. Asterisks indicate significance of two-tailed unpaired t test. * P<0.05, ** P<0.01, *** P<0.001. n.s. not statistically significant.

In *Med23^flox/flox^;Lck-Cre* mice with efficient deletion in the thymus (Figure S1B and S1C), the proportions of CD4 CD8 double negative (DN), double positive (DP) and single positive cells were normal (Figures 1A and S2A). While the *Lck-Cre* transgene itself caused a modest reduction in thymocyte number, *Med23^flox/flox^;Lck-Cre* mice had similar thymocyte counts to mice with the *Lck-Cre* transgene alone (Figure 1B).

**Figure 2:**
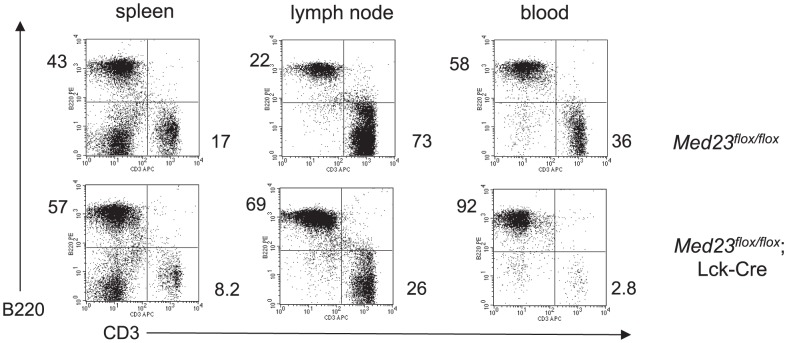
Mice with efficient deletion of *Med23* exhibit decreased T-cell numbers in the peripheral lymphoid organs. Flow cytometric analysis of spleen, lymph node and blood from *Med23^flox/flox^* and *Med23^flox/flox^;Lck-Cre* mice showing proportions of CD3^+^ T-cells and B220^+^ B-cells. Flow cytometry from mouse shown is representative of phenotype seen in mice with greater than 95% deletion of *Med23* in the thymus.

To further assess the MED23 null T-cell populations in the thymus and peripheral lymphoid organs, we utilized an *eYFP* transgene that is turned on by Cre-mediated recombination [34] and marks cells where the *Lck-Cre* transgene is expressed. We then determined the percentage of YFP^+^ cells in the thymus, spleen, lymph nodes and blood of *Med23^+/+^;Lck-Cre;YFP* (control) and *Med23^flox/flox^;Lck-Cre;YFP* mice by flow cytometry (Figure 1C shows average percentage of YFP^+^ cells normalized to YFP^+^ DP thymocytes for each mouse; see Table S1 for unnormalized YFP percentages). In *Med23^+/+^;Lck-Cre;YFP* control mice the percentage of YFP^+^ T-cells remained constant between the thymus and peripheral lymphoid organs (Figure 1C, Table S1, *N* = 2 control mice). However, for *Med23^flox/flox^;Lck-Cre;YFP* mice, the percentage of YFP^+^ (*Med23* null) T-cells compared to DP thymocytes, was significantly reduced in spleen, lymph nodes and blood (Figure 1C, Table S1, *N* = 7 mutant mice; *P* = 0.0035 to <0.0001). For both *Med23^+/+^;Lck-Cre;YFP* and *Med23^flox/flox^;Lck-Cre;YFP* mice the percentage of YFP^+^ DP thymocytes was comparable to the percentage of YFP^+^ CD4 and CD8 single positives (Figure 1C, Table S1). This indicates that loss of MED23 does not result in a block in thymocyte maturation from double positive to single positive cells. The DN thymocyte compartment had a reduced percentage of YFP^+^ T-cells for both *Med23^+/+^;Lck-Cre;YFP* and *Med23^flox/flox^;Lck-Cre;YFP* mice (Figure 1C), reflecting that the *Lck-Cre* transgene does not turn on until the DN4 stage of development [33],[41]. Consistent with this data, in *Med23^flox/flox^;Lck-Cre* mice with very high deletion in the thymus, a decrease in the ratio of T-cells (CD3^+^) to B-cells (B220^+^) in spleen, lymph node and blood compared to *Med23^flox/flox^* control mice was evident (Figure 2, Figure S2A). In *Med23^flox/flox^;Lck-Cre* mice with more modest *Med23* deletion in the thymus this deficit in peripheral T-cell numbers was not seen, as the number of undeleted T-cells in the thymus was sufficient to completely populate the periphery (Figure S2A–C, compare mouse with poor deletion (#2) to mice with high deletion (#1,3 and 4)).

### MED23 null T-cells do not display increased apoptosis

**Figure 3:**
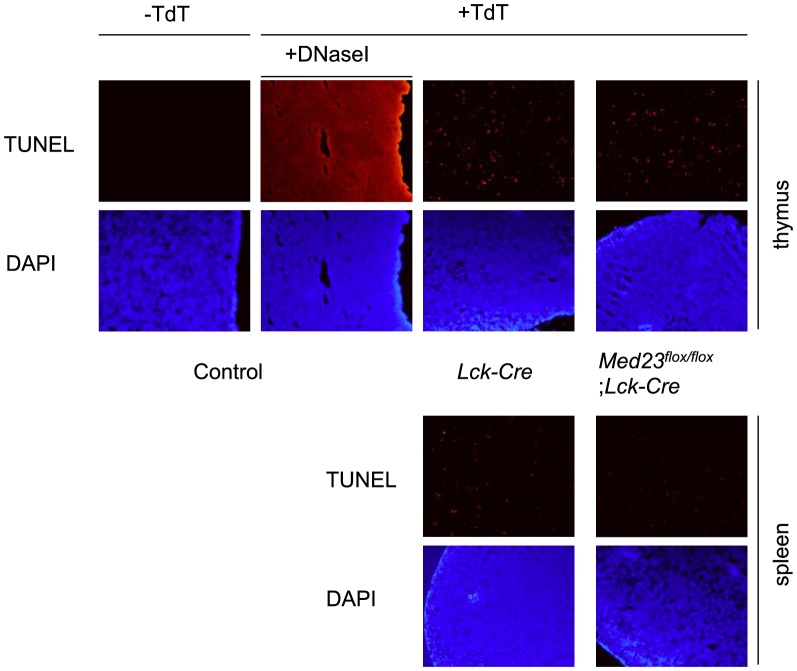
*Med23^flox/flox^;Lck-Cre* T-cells do not display increased apoptosis. TUNEL assay was performed on cryosections from thymus and spleen of *Med23^flox/flox^; Lck-Cre* and *Lck-Cre* control mice. A control section was treated with DNaseI prior to TUNEL staining (+TdT) as a positive control, and a cryosection that was not stained (−TdT) serves as a negative control.

The deficit of MED23 null T-cells in the peripheral lymphoid organs, led us to investigate whether there was an increased rate of T-cell apoptosis in *Med23^flox/flox^;Lck-Cre* mice compared to controls. Increased apoptosis *in situ* was not apparent as TUNEL assays showed no difference between *Med23^flox/flox^;Lck-Cre* and *Med23^+/+^;Lck-Cre* mice in cryosections from either thymus or spleen (Figure 3).

### MED23 null thymocytes proliferate normally in response to activation, but display abnormal gene expression

**Figure 4:**
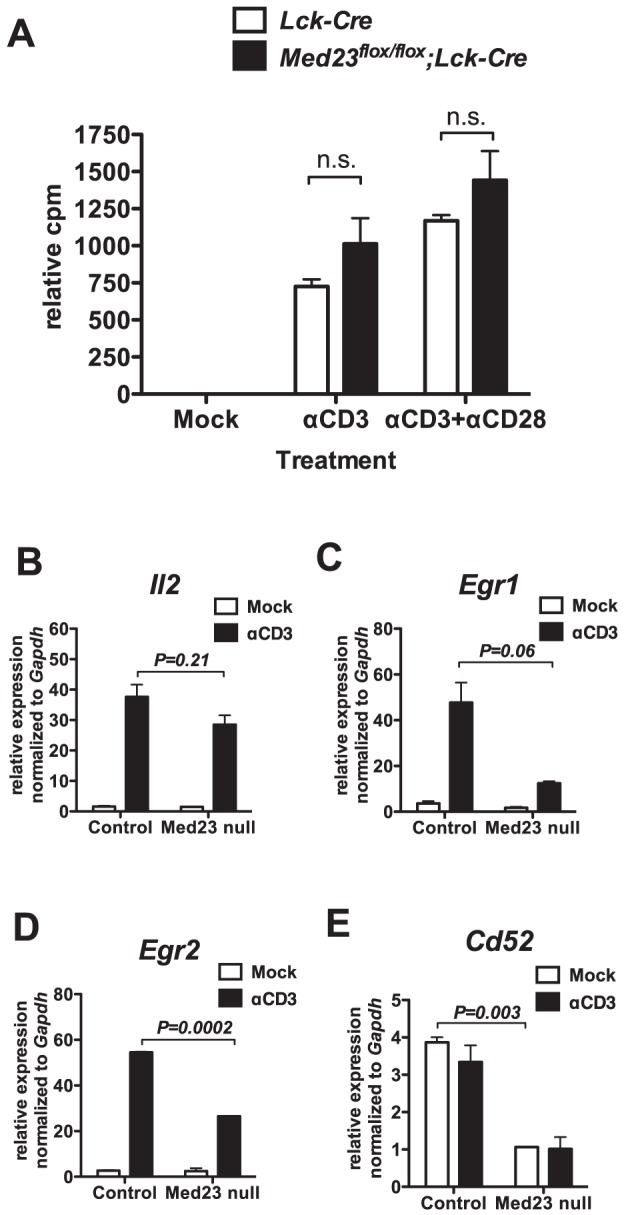
MED23 null thymocytes proliferate normally in response to stimulation, but have abnormal gene expression. **A** Total thymocytes were cultured in the presence of plate bound αCD3 and αCD28 antibodies (as indicated) for 44 hrs, then allowed to incorporate ^3^H thymidine for 18 hrs. Counts per minute (CPM) were normalized to the *Lck-Cre* Mock counts for each experiment. N = 2–3, Mean +/− SEM. Statistical analysis shown is Tukey post-test following one way ANOVA, n.s. not statistically significant. **B–E**, *Il2* (**B**), *Egr1* (**C**), *Egr2* (**D**), and *Cd52* (**E**) mRNA expression analyzed by qRT-PCR in *Med23^+/+^;Lck-Cre* (control) and *Med23^flox/flox^;Lck-Cre* (Med23 null) total thymocytes rested 4 hours after harvest, then mock treated or treated 3 hours *ex vivo* with immobilized αCD3. *Med23^flox/flox^;Lck-Cre* thymocytes showed greater than 90% deletion. N = 2, Mean +/− SEM. *P* values from two-tailed t-tests between αCD3 (B–D) or Mock (E) treated samples.

The ERK/MAPK pathway is also known to be important in the activation of T-cells including their proliferative response [24],[29]. We next harvested total thymocytes from *Med23^+/+^;Lck-Cre* and *Med23^flox/flox^;Lck-Cre* mice and cultured them in the presence of plate-bound αCD3 antibody, or antibodies against both CD3 and CD28 (Figure 4A). We found that proliferation in response to αCD3 alone, or αCD3 and αCD28 together, was not significantly different in MED23 null thymocytes compared to controls (Figure 4A).

We next activated *Med23^+/+^;Lck-Cre* and *Med23^flox/flox^;Lck-Cre* total thymocytes for 3 hours by exposure to plate-bound αCD3 antibody, followed by isolation of mRNA. *Il2* mRNA increased in response to TCR activation as expected and this induction was comparable in MED23 null and control thymocytes (Figure 4B). The known MED23 target genes, *Egr1* and *Egr2* were induced by T-cell activation and had reduced expression in MED23 null compared with control thymocytes (Figure 4C,D; *P* = 0.06 and 0.0002, *N* = 2). Microarray analysis revealed 26 genes that were induced at least two fold by αCD3 (Table S2). Seven of these genes displayed αCD3 inducible expression that was at least twofold lower in *Med23^flox/flox^;Lck-Cre* compared with control thymocytes (*Med23^+/+^;Lck-Cre* and *Med23^flox/flox^*), including *Egr1*, *Egr2*, *Cd5*, *Cd6*, *Nab2*, *Pcyt1a* and *Ier2*.

### KLF2 expression is decreased in *Med23* null single positive thymocytes

Interestingly, microarray analysis of the mock-treated thymocytes revealed that *Cd52* (CAMPATH-1) mRNA expression was decreased in MED23 null thymocytes compared to controls (Table S3). This defect in *Cd52* expression was confirmed by qRT-PCR (Figure 4E; *P* = 0.003, *N* = 2). *Cd52* has previously been shown to be a target of the transcription factor KLF2 [42] and there are distinct similarities between the phenotype of *Med23* null T-cells, and T-cells lacking *Klf2* (*Lklf*) or the KLF2 target gene, *S1pr1* (*Edg1*). All three of these mutant mice retain relatively normal thymic development, but the mutant T-cells have a reduced ability to populate the peripheral lymphocytic organs [36],[43] [46].

**Figure 5:**
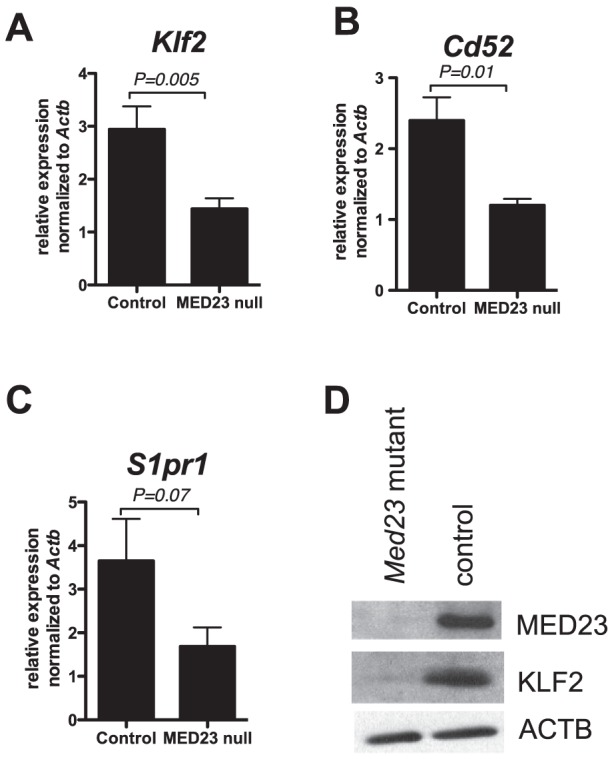
MED23 null single positive thymocytes express decreased KLF2. **A–C**, *Klf2* (**A**), *Cd52* (**B**) and *S1pr1* (**C**) mRNA expression in CD4^+^ single positive thymocytes; YFP^+^ *Med23^+/+^;Lck-Cre;YFP* or *Med23^+/+^;Lck-Cre* (control) and YFP^+^ *Med23^flox/flox^;Lck-Cre;YFP* or *Med23^flox/flox^;Lck-Cre* where deletion was greater than 90% (MED23 null) thymocytes. Two-tailed t test; N = 4–8; Mean +/− SEM. **D**, Western blot of MED23, KLF2 and ACTB (loading control) in whole cell extracts from YFP^+^ CD4 SP SP thymocytes from *Med23^+/+^;Lck-Cre;YFP* (control) and *Med23^flox/flox^;Lck-Cre;YFP* (mutant) mice.

In the thymus, KLF2 is expressed predominantly in single positive (SP) thymocytes [36], so we isolated CD4 SP thymocytes from *Med23^+/+^;Lck-Cre* and *Med23^flox/flox^;Lck-Cre* mice and measured gene expression. We found that expression of *Klf2* mRNA was significantly downregulated in MED23 null CD4 SP thymocytes (Figure 5A; *N* = 8; *P* = 0.005) as was expression of the KLF2 target gene, *Cd52* (Figure 5B; *N* = 4; *P* = 0.01). The KLF2 target gene, *S1pr1* [42] also tended to be expressed lower in MED23 null CD4 SP thymocytes (Figure 5C; *N* = 8; *P* = 0.07). Importantly, KLF2 protein levels were also lower in MED23 null CD4 SP thymocytes compared to controls (Figure 5D).

### *Med23^Δflox/Δflox^* MEFs also have decreased *Klf2* expression

**Figure 6:**
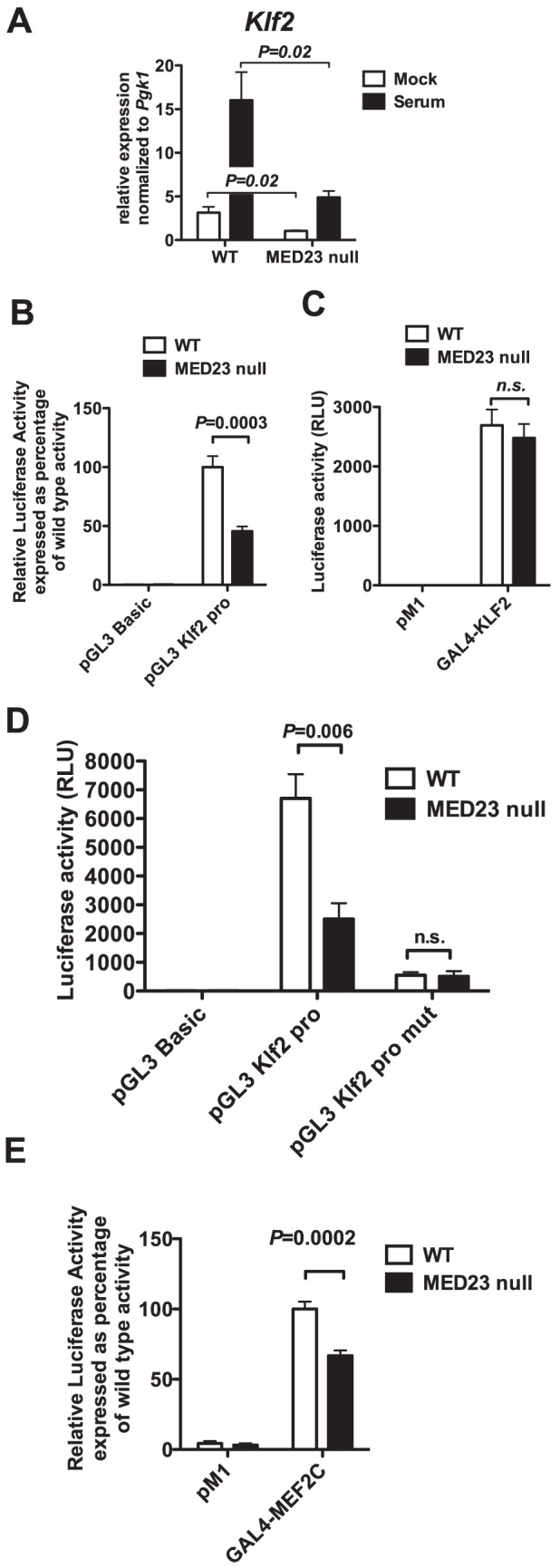
MEFs lacking MED23 show altered *Klf2* expression. **A**, *Klf2* mRNA expression by qRT-PCR in *Med23^+/+^* (WT) and *Med23^Δflox/Δflox^* (MED23 null) MEFs treated with Cre adenovirus, then three days later starved overnight and treated with serum for 1 hour. *N* = 4, mean +/− SEM, two-tailed t test, *P* = 0.02 for comparison of either mock or serum-treated samples. **B**, Activity of a luciferase reporter construct driven by the *Klf2* promoter (pGL3-*Klf2*-pro) in WT and MED23 null MEFs (*N* = 6, mean +/− SEM, two-tailed t test, *P* = 0.0003). **C**, Activity of a GAL4-KLF2 fusion construct containing aa 1–88 of KLF2 in WT and MED23 null MEFs (*N* = 2, triplicates, mean +/− SEM). **D**, Mutation of a MEF2 site in the *Klf2* promoter construct (pGL3-*Klf2*-pro-mut) dramatically decreases activity of the construct (*N* = 2, duplicates, mean +/− SEM). **E**, A GAL4-MEF2C fusion construct is dependent upon MED23 for full activity (*N* = 4–8, mean +/−SEM, two-tailed t test *P* = 0.0002). pGL3 Basic and pM1 are empty luciferase and GAL4 DNA binding domain (DBD) vectors respectively. n.s., not statistically significant.

Using *Med23^flox/flox^* MEFs treated with adenovirus expressing Cre recombinase (Figure S3), we sought to further elucidate the regulation of *Klf2* expression by MED23. *Klf2* mRNA in MEFs is induced in response to serum and we found that both the basal (with 0.1% serum) and serum-induced (10% serum) expression of *Klf2* was attenuated by loss of MED23 (Figure 6A; *N* = 4, *P* = 0.02).

We next examined the activity of a luciferase reporter driven by the *Klf2* promoter (pGL3-*Klf2*-pro) and found that its activity was significantly decreased in MEFs lacking MED23 (Figure 6B; *N* = 6, *P* = 0.0003). Since there is evidence that expression of KLF2 target genes, *Cd52* and *S1pr1*, is also affected by MED23 loss, we examined the activity of a construct fusing the GAL4 DNA binding domain (DBD) to the first 88 amino acids of KLF2, which includes the activation domain (GAL4-KLF2). We found that in contrast to the effect of MED23 on *Klf2* expression, MED23 loss does not adversely affect KLF2-dependent transactivation (Figure 6C).

It has previously been reported that MEF2 binds to the *Klf2* promoter, and that this binding is required for MAPK/ERK5-dependent expression of *Klf2* in response to shear stress [39],[47]. MEF2C is also reported to be a serum-responsive transactivator [48], further suggesting it contributes to the serum-dependent expression of *Klf2* (Figure 6A). We found that mutation of this MEF2 binding element in our *Klf2* promoter luciferase construct dramatically decreased its activity in both wild type and MED23 null MEFs (Figure 6D). The remaining luciferase activity after mutation of the MEF2 site was MED23-independent as the activity of the mutated construct was the same in wild type and MED23 null MEFs (Figure 6D). Based on this result, we tested the transactivation capacity of a GAL4 DBD full length MEF2C fusion construct (GAL4-MEF2C) and found that it was decreased by about 40 percent in MEF23 null MEFs (Figure 6E, *N* = 4–8, *P* = 0.0002). This suggests that MED23 could mediate *Klf2* expression by providing coactivation function to MEF2 transcription factors.

## Discussion

In this study, MED23 was found to have a rather specific role in T-cell gene expression and development/function, even though it is a subunit of the global coactivator complex Mediator. With regard to T-cell biology, we found that MED23 is necessary for T-cells to populate the periphery. We also showed that *Med23* is required for the expression of target genes, *Egr1* and *Klf2*, which have both been previously shown to reduce T-cell numbers outside the thymus when mutated (Figures 3 and 4) [36],[45],[49],[50]. MED23 is also required to maintain KLF2 protein levels and KLF2-target gene expression (*Cd52*), indicating that the reduction in *Klf2* gene expression has functional consequences for the cells (Figure 5). Finally, the transactivation function of the MEF2 family of transcription factors includes a MED23-dependent component and may contribute to *Klf2* expression (Figure 6).

### The phenotype of *Med23^flox/flox^;Lck-Cre* mice is distinct from the T-cell phenotypes of *Elk1^−/−^* and *Elk4^−/−^* mice

Mice null for ELK1, an ETS family transcription factor that is known to utilize Mediator via the MED23 subunit [20], have no obvious phenotype [51]. However, knockout of fellow ETS family member ELK4 (SAP1) results in defective positive selection of T-cells in the thymus and double-knockout of both *Elk1* and *Elk4* enhances this defect [52]. Despite evidence that both ELK1 and to a lesser degree, ELK4 require the MED23 subunit for coactivation by Mediator [17],[20], we did not see a defect in positive selection. Several reasons for this phenotypic disconnect are possible. First, the timing of *Med23* deletion with *Lck-Cre* (DN4) may be too late to influence positive selection (i.e. MED23 protein may still be present during the critical events). In the future, use of a different Cre transgene that produces earlier deletion in the T-cell compartment could help answer this question. Second, the level of MED23-dependent coactivation provided to ELK4 in T-cells may be insufficient to affect positive selection. In this regard, our own and other studies have shown that loss of MED23 can have the effect of decreasing, but not ablating expression of target genes [17],[20]. A third possibility is that the subset of ELK1/ELK4 target genes that are MED23-dependent in T-cells are not those that are required for normal positive selection. Other models support this possibility, as we have found that both the transcription factors CREB and HIF require the coactivators CBP and p300 for expression of some, but not all of their target genes [5],[8],[12],[53]. Likewise, it has been shown that cellular context influences the requirement for MED23 for the expression of target genes in MEFs compared with ES cells, even in response to the same stimulus [17].

### MED23 target genes *Klf2* and *Egr1* impact the number of peripheral T-cells in knockout mouse models

We have shown that conditional deletion of *Med23* in the T-cell lineage of mice produces T-cells with a decreased ability to populate the periphery (Figures 1 and 2). It seems likely that this defect may be linked to a decrease we observe in *Klf2* mRNA and KLF2 protein levels in single positive thymocytes, since *Klf2* deletion in T-cells results in a similar, albeit more complete loss of peripheral T-cells [36],[45],[50].

The reduced expression of *Egr1* in MED23 null T-cells may also play a role in their decreased ability to populate the peripheral organs. *Egr1* has been shown to be necessary for the survival of recent thymic emigrants in the periphery, although this phenotype was seen only in TCR transgenic mice [49]. Interestingly, other phenotypes associated with *Egr1* deletion, such as increased thymocyte number [54], were not seen in our study. One possible explanation is that deletion of *Med23* during thymic development (DN4 stage for the *Lck-Cre* transgene used in this study) [41] results in a milder phenotype than would be seen if MED23 were absent from the start of thymic development.

Our data show that MED23 is required for full expression of the endogenous *Klf2* gene in both T-cells and MEFs (Figures 5 and 6). Our *in vitro* data suggest that MEF2 transcription factors may play a role in *Klf2* expression in MEFs, as mutation of a MEF2 binding site in a *Klf2* promoter luciferase reporter construct drastically reduces the activity of the construct (Figure 6D). Likewise, the transactivation function of a GAL4-MEF2C fusion construct is significantly reduced in MED23 null MEFs (Figure 6E), suggesting that MEF2 family members may utilize MED23-dependent mechanisms of coactivation. *Klf2* has been previously reported to be induced in a MAPK/ERK5-dependent manner by shear stress in endothelial cells through the transcription factor MEF2 [47]. However, knockout of ERK5 in mouse T-cells produces no deficit in thymocyte development or peripheral T-cell numbers, although there was impaired induction of *Klf2* mRNA in response to TCR signaling [55] [56]. MEF2C expression has been shown to be critical for commitment to the lymphoid lineage, but does not seem to be necessary for thymocyte development [57]. Thus, whether MEF2 is the key regulator of *Klf2* in T-cells remains to be established.

### Cell type context in Mediator MED23 subunit utilization

The MED23 subunit was originally implicated as critical for recruitment of the Mediator complex by the transcription factor ELK1, as well as the adenovirus E1A protein [20]. More recently, it has been shown that in at least some gene contexts, the MED23 subunit also appears to be important for the transition from paused to Ser2 phosphorylated elongating RNA Polymerase II via the recruitment of positive elongation factor b (P-TEFb) [58]. In this scenario, the Mediator complex is recruited to target gene promoters equally in the presence or absence of MED23; however, MED23 is required for gene transcription to occur [58]. It is not clear yet what context determines which coactivation mechanism(s) MED23 can contribute in specific cell types or at certain promoters. Our study contributes to the findings that the MED23 subunit of Mediator is required for the expression of a narrow, but important subset of genes in a range of cell types [17],[20]–[22],[58].

Here we described a new *Med23* conditional knockout mouse and its utility in studying this Mediator subunit *in vitro* and *in vivo*. Using this model, we established that MED23 has a rather specific biological role in the context of T-cells, and is critical for the expression of the T-cell master regulatory transcription factor, KLF2.

## Supporting Information

Figure S1 Conditional allele of *Med23* results in a null allele after cre-mediated recombination. **A**, Targeting strategy for *Med23* conditional knockout mice. **B,C**, Southern (**B**) and western (**C**) showing efficient deletion of *Med23* floxed alleles and MED23 protein in thymus from a *Med23^flox/flox^;Lck-Cre* mouse. Western blot performed using an antibody raised against amino acids 906–925.
(PDF)

Figure S2 Mice with efficient Med23 deletion have reduced peripheral T cell numbers. **A**, Scatter plots showing CD4 vs CD8 (thymus) or B220 vs CD3 (spleen and blood) for one *Med23^+/+^;Lck-Cre* control mouse and four *Med23^flox/flox^; Lck-Cre* mice. **B**, Southern blot showing that *Med23^flox/flox^; Lck-Cre* mice #1, 3 and 4 have efficient recombination of the floxed alleles while #2 has less than 50% recombination. **C**, Western blot confirming MED23 protein loss in efficiently deleted *Med23^flox/flox^; Lck-Cre* mice.
(PDF)

Figure S3 *Med23^Δflox/Δflox^* MEFs treated with adenovirus expressing Cre recombinase show efficient deletion of MED23. **A**. Semiquantitative PCR of genomic DNA from Cre adenovirus treated wild type (*Med23^+/+^*) and *Med23^Δflox/Δflox^* MEFs demonstrating efficient recombination of the conditional *Med23* allele. Bands for the wild type (WT), conditional (flox) and recombined conditional (Δflox) alleles are indicated. **B**. Western blot of Cre-adenovirus treated wild type and *Med23^Δflox/Δflox^* MEFs showing loss of MED23 protein. Non-specific band shown as loading control.
(PDF)

Table S1 YFP^+^ percentages for each cell population in each mouse that were used to calculate the average normalized YFP^+^ percentages shown in Figure 1C.
(XLSX)

Table S2 Genes induced at least two fold by *ex vivo* αCD3 treatment of total thymocytes from control (*Lck-Cre* and *Med23^flox/flox^*) mice based on microarray analysis.
(XLSX)

Table S3 Genes expressed at least two fold higher in *ex vivo* mock-treated total thymocytes from control (*Lck-Cre* and *Med23^flox/flox^*) mice compared with *Med23^flox/flox^; Lck-Cre* mice based on microarray analysis.
(XLSX)
