# Supplementary figures and images for: T-Cells Null for the MED23 Subunit of Mediator Express Decreased Levels of KLF2 and Inefficiently Populate the Peripheral Lymphoid Organs
Source: PLoS One. 2014 Jul 23;9(7):e102076. doi: 10.1371/journal.pone.0102076 (PMC4108324; doi:10.1371/journal.pone.0102076)

**Figure S1**

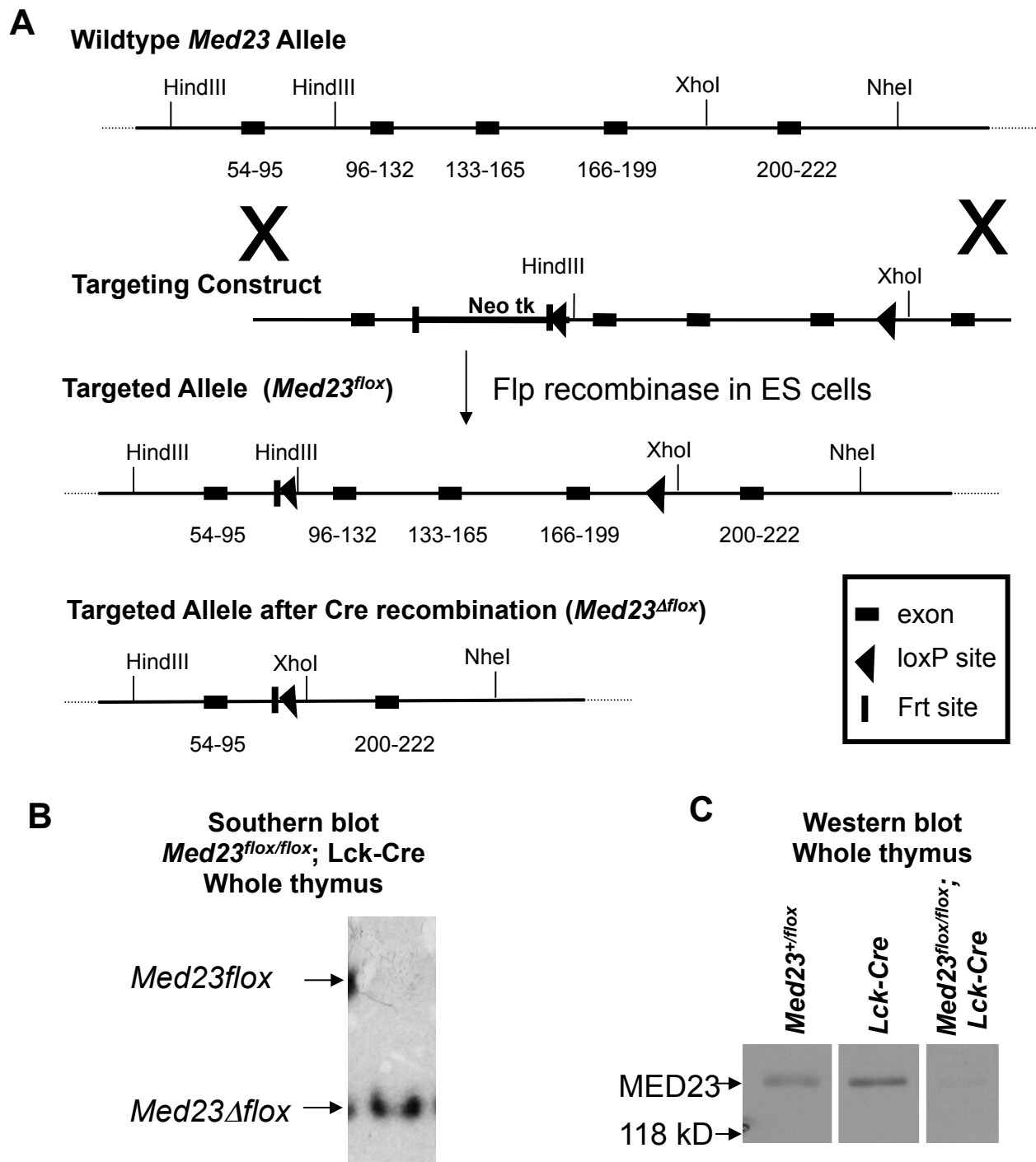

Supplement: Figure S1 — Conditional allele of Med23 results in a null allele after cre-mediated recombination. A, Targeting strategy for Med23 conditional knockout mice. B,C, Southern (B) and western (C) showing efficient deletion of Med23 floxed alleles and MED23 protein in thymus from a Med23flox/flox;Lck-Cre mouse. Western blot performed using an antibody raised against amino acids 906–925. (PDF) [file pone.0102076.s001.pdf]

**Figure S2**

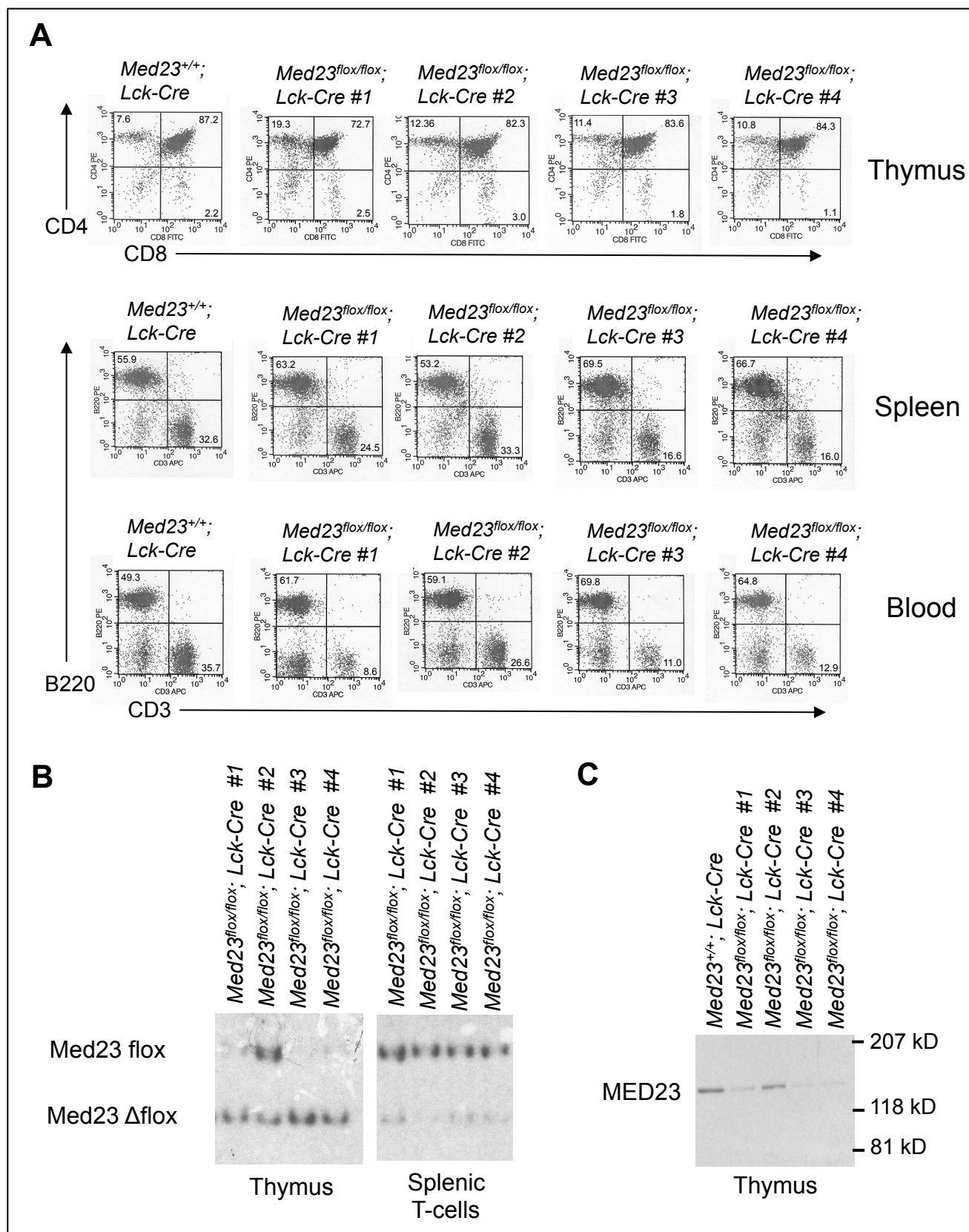

Supplement: Figure S2 — Mice with efficient Med23 deletion have reduced peripheral T cell numbers. A, Scatter plots showing CD4 vs CD8 (thymus) or B220 vs CD3 (spleen and blood) for one Med23+/+;Lck-Cre control mouse and four Med23flox/flox; Lck-Cre mice. B, Southern blot showing that Med23flox/flox; Lck-Cre mice #1, 3 and 4 have efficient recombination of the floxed alleles while #2 has less than 50% recombination. C, Western blot confirming MED23 protein loss in efficiently deleted Med23flox/flox; Lck-Cre mice. (PDF) [file pone.0102076.s002.pdf]

Figure S3

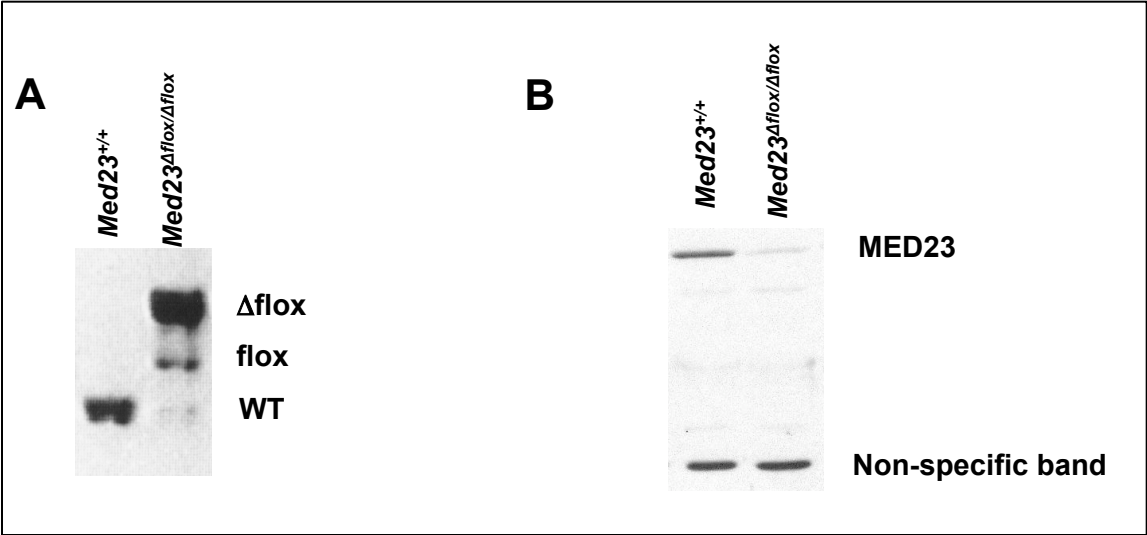

Supplement: Figure S3 — Med23Δflox/Δflox MEFs treated with adenovirus expressing Cre recombinase show efficient deletion of MED23. A. Semiquantitative PCR of genomic DNA from Cre adenovirus treated wild type (Med23+/+) and Med23Δflox/Δflox MEFs demonstrating efficient recombination of the conditional Med23 allele. Bands for the wild type (WT), conditional (flox) and recombined conditional (Δflox) alleles are indicated. B. Western blot of Cre-adenovirus treated wild type and Med23Δflox/Δflox MEFs showing loss of MED23 protein. Non-specific band shown as loading control. (PDF) [file pone.0102076.s003.pdf]
